# Implementation of a Knowledge Management System in Mental Health and Addictions: Mixed Methods Case Study

**DOI:** 10.2196/39334

**Published:** 2023-02-06

**Authors:** Jill Chorney, Debbie Johnson Emberly, Jennifer Jeffrey, Amos Hundert, Onur Pakkanlilar, Sabina Abidi, Alexa Bagnell, Maureen Brennan, Leslie Anne Campbell, Sharon Clark, Kristina Bradley, Olivia Ross

**Affiliations:** 1 Department of Psychiatry Dalhousie University Halifax, NS Canada; 2 Mental Health and Addictions Program IWK Health Halifax, NS Canada; 3 Emergency Medical Care Inc Halifax, NS Canada; 4 Department of Community Health and Epidemiology Dalhousie University Halifax, NS Canada

**Keywords:** mental health, knowledge management, information, technology, capacity building

## Abstract

**Background:**

Mental health and addictions (MHA) care is complex and individualized and requires coordination across providers and areas of care. Knowledge management is an essential facilitator and common challenge in MHA services.

**Objective:**

This paper aimed to describe the development of a knowledge management system (KMS) and the associated processes in 1 MHA program. We also aimed to examine the uptake and use, satisfaction, and feedback on implementation among a group of pilot testers.

**Methods:**

This project was conducted as a continuous quality-improvement initiative. Integrated stakeholder engagement was used to scope the content and design the information architecture to be implemented using a commercially available knowledge management platform. A group of 30 clinical and administrative staff were trained and tested with the KMS over a period of 10 weeks. Feedback was collected via surveys and focus groups. System analytics were used to characterize engagement. The content, design, and full-scale implementation planning of the KMS were refined based on the results.

**Results:**

Satisfaction with accessing the content increased from baseline to after the pilot. Most testers indicated that they would recommend the KMS to a colleague, and satisfaction with KMS functionalities was high. A median of 7 testers was active each week, and testers were active for a median of 4 days over the course of the pilot. Focus group themes included the following: the KMS was a solution to problems for staff members, functionality of the KMS was important, quality content matters, training was helpful and could be improved, and KMS access was required to be easy and barrier free.

**Conclusions:**

Knowledge management is an ongoing need in MHA services, and KMSs hold promise in addressing this need. Testers in 1 MHA program found a KMS that is easy to use and would recommend it to colleagues. Opportunities to improve implementation and increase uptake were identified. Future research is needed to understand the impact of KMSs on quality of care and organizational efficiency.

## Introduction

### Mental Health and Addictions Care

Mental health and addictions (MHA) care is complex and individualized and requires coordination across providers and areas of care [1]. To provide the highest quality care, MHA care providers require access to a wide range of knowledge and information sources including up-to-date evidence, tools to translate evidence into clinical practice, learning resources, and information on available supports (eg, community resources and expert consultation). When working in an organization, MHA care providers also need access to current organizational policies, practices, and procedures and need to share this access with colleagues and administrative staff. Getting the right information to the right people at the right time to facilitate high-quality care is a significant challenge, especially in an era of increasing knowledge production and continually updated policies, procedures, and processes.

### Mental Health and the Promise of Knowledge Management

Knowledge management is a field that holds significant promise for MHA care. Broadly speaking, knowledge management refers to the systematic and coordinated creation, sharing, and application of knowledge to promote innovation and add value to organizations [2]. Knowledge management has increasingly been facilitated by technological solutions, often called knowledge management systems (KMSs) [3,4]. KMS solutions vary, but many currently available products include features, such as advanced search and retrieval capability, embedded content management workflows, and the use of artificial intelligence to identify connected content and suggest content to users. In several research studies, technology-supported knowledge management has been associated with improved organizational performance including increased innovation and organizational success [5-7].

Although knowledge management is a recognized need across sectors, KMS solutions have been relatively underutilized in health care and mental health care specifically [5,8,9]. Attending to the development and implementation processes is important for the ultimate success of KMS projects [10]. Factors such as the quality of the technology, the quality of the information, system usability, motivation of testers, and support from leaders have been found to influence the likelihood of KMS uptake and positive outcomes [5,10-12]. Knowledge translation and implementation frameworks also highlight the importance of engaging end testers in the development of innovations or KMS tools [12].

### Current Project

To date, there are few descriptions of the development and implementation of KMS solutions in MHA care, and there are no evaluations of the uptake of these solutions. Providing an in-depth description of the process and outcomes of introducing a KMS within an MHA setting can guide future research and practice. This paper describes a continuous quality-improvement project to develop and pilot-test a KMS and its implementation in 1 MHA program.

The objectives of this study were to describe the development of a KMS and associated processes in 1 MHA program and to examine the uptake and use, satisfaction, and feedback on implementation among a group of pilot testers.

## Methods

### Context

This project was conducted within the MHA program at Izaak Walton Killam Health, Halifax, Nova Scotia, Canada. The MHA program provides family-centered mental health, addictions, and forensic services for children and adolescents aged up to 19 years and reproductive mental health services for perinatal individuals. The MHA program has local, provincial, and regional mandates, with services located in 9 different communities. Services include 5 inpatient units, 8 psychiatry-led specialty clinics, 5 intensive day treatment services, and 15 community-based services (offered in MHA clinics, schools, and other community locations). The program has approximately 450 interdisciplinary care providers (eg, nurses, occupational therapists, social workers, recreation therapists, teachers, psychiatrists, and psychologists) and administrative staff (eg, booking and registration clerks, administrative assistants, and ward clerks). In the fiscal year 2021, the MHA program provided care to 5844 clients, conducted 50,440 outpatient visits, and had 330 inpatient admissions.

The MHA program has been engaged in focused work with the vision of ensuring that all children, youth, and families (including perinatal individuals) across Nova Scotia receive the *right care, at the right time, by the right person, and in the right place*. The program had a wealth of clinical and administrative knowledge that would enable this vision, but materials conveying this knowledge (eg, training presentations, manuals, client resources, standard administrative processes, and forms) were stored in siloed and fragmented locations without a formal management process. This led to inefficiencies in searching, finding, and sharing content; the risk of out-of-date content use; and unequal access to knowledge and resources across diverse providers and services in the MHA program. The lack of systemic knowledge management was a challenge not only for day-to-day work but also for quality-improvement efforts. In 2020, MHA leaders identified knowledge management as a priority and began a process to operationalize a commercially available KMS to address this need.

### KMS Development

#### Procurement

An institutional procurement process was used to select a commercial KMS vendor. Procurement included a formal request for proposals that outlined project requirements, as well as organizational and provincial standards. Representatives from the organization’s Privacy and Information Technology departments were engaged to ensure that the KMS was integrated with organizational standards. Institutional procurement processes resulted in the selection of an electronic KMS platform (Shelf), which provided advanced search capabilities, artificial intelligence–driven content suggestions, automated content and feedback management processes, and robust user analytics.

#### Governance and Stakeholder Engagement

The KMS project leads (a clinical lead and a project management lead) had primary responsibility for the project and worked under the guidance of a steering committee that included key MHA program leaders. The vendor provided ongoing support throughout the development and implementation process, but the decision-making authority rested with the project leads and steering committee. To facilitate end-user engagement, a group of 12 clinical co-designers were recruited to work closely with the project leads to identify relevant clinical content, inform the information architecture, and consult on the implementation plan. Clinical co-designers were self-selected and represented various care areas and professional disciplines in the program. Additional subject matter experts were identified and engaged ad hoc by the project leads. An evaluation team, which included clinical staff, designed the methodology and collected and analyzed data from the pilot study. The co-designer and evaluation groups reported to the steering committee via project leads.

Stakeholders were engaged throughout the project. This included routine follow-up with testers (ie, asking for recommended sources of content when gaps were identified), relationship building with additional subject matter experts (ie, addressing frequently asked questions about content sharing), continued consultation with co-designers (ie, reviewing pilot results for interpretation and planning), additional steering committee guidance (ie, approving pursual of specific proprietary content sources), and manager engagement (ie, scoping additional operational content, refining implementation plan, and timing).

#### Content Identification and Migration

The potential content for the KMS was scoped via a review of current storage locations (ie, organization-wide learning sites and program-wide and team-specific shared drives). Co-designers and subject matter experts identified additional content that is commonly used in clinical practice and capacity-building initiatives. To ensure that external content was consistent with current evidence and best practices, content was vetted by the clinical project lead, co-designers, or subject matter experts before migrating to the KMS.

Content identified included resources developed both internally (ie, developed by staff in the organization) and externally (ie, copyright was owned by individuals or groups outside the organization). Given the high quality of external content and gaps that would otherwise be evident if limited to only internal content, a decision was made to include external content where possible. On the basis of consultation with the institutional risk management and legal team, an approach was developed to seek expressed consent for distribution from the copyright owner or to confirm permission to distribute content for noncommercial purposes in publicly available terms and conditions. Permissions were confirmed and tracked by the project management lead. If content was freely available on the web but permission to distribute was not available or unclear, web links (rather than direct uploads) were included.

#### Information Architecture

A draft of the information architecture (ie, library and folder structure, preliminary tags, and categories of document types) was developed based on a rough thematic analysis of the identified content and by considering the values and directions of the MHA Program. Decisions about information architecture aimed to build common practices, highlight connections, and develop a shared language across testers. In this way, decisions explicitly attempted to avoid reinforcing existing silos of care (eg, creating separate libraries for different areas of the program). Decisions about the information architecture were also guided by a formulation approach (eg, presenting problems, transdiagnostic mechanisms of change, and biopsychosocial contributors) rather than strict diagnostic categories (ie, disorder specific) or treatment modality (eg, cognitive behavioral therapy). Where possible, preference was given to the use of inclusive and community-driven language (eg, neurodiversity) rather than medical terminology (eg, neurodevelopmental disorders). Operational definitions of content in each folder were also provided to maintain the consistency and cohesiveness of materials within each folder, as well as to guide the placement of new content. The pilot version of the information architecture included 6 high-level libraries: KMS Resources (tip sheets and terms of use), About MHA Program (service descriptions and referral forms), Community Resources (information about community partner organizations and programs), Group Resources (information and materials used to facilitate therapeutic groups), Standard Work Dashboard (clinical documentation forms and standard processes), and Clinical Resources (handouts, worksheets, videos, and clinical training materials to use in care provision). Tags were used to link content across folders and to allow for more nuanced and diverse search terms. Approximately 100 tags were identified, covering a range of commonly used terms (eg, concurrent disorders and cognitive behavioral therapy). Document types were included as categories (eg, client worksheet, standard work, fillable form, and video). Project leads, co-designers, and subject matter experts migrated content onto the KMS and iteratively refined the folder structure to include new content.

#### Content Management Processes

Content vetting and management processes were developed to outline the responsibilities and review cycles. The platform was configured to provide permissions for uploading and vetting content to project leads and to select subject matter experts. Terms of use were also developed in consultation with organizational legal services. Terms of use included an agreement to use content in the context of users’ professional training and roles, not to distribute content outside of personal use in users’ practice (ie, content can be shared with clients but not other organizations), and no commercial use of content.

### Pilot Evaluation

A mixed methods pre-post evaluation was conducted over 10 weeks (June-August 2021).

#### Ethical Considerations

The project was assessed by the IWK Health Research Ethics Board to be a quality-improvement initiative and thus was exempted from human subject research ethics review. Testers provided consent to participate in this project for internal quality-improvement purposes and were informed of the secondary use of data for the purposes of this report. Managers supported testers to participate in KMS training, complete surveys, and participate in focus groups during work hours. No additional compensation was provided for the secondary use of data. All data reported here are deidentified and reported at the aggregate level.

#### Participant Recruitment

Testers were recruited via email sent to all staff of the MHA program (approximately 400 interdisciplinary staff and physicians) through word of mouth, and individually targeted approaches by managers or KMS project leads. Recruitment materials included information about the purpose of the project and specifically requested participation from those with varied interests and experience with technology. Purposeful sampling was used to ensure representation from a range of roles (ie, clinicians and administrative staff), disciplines (eg, psychiatry, social work, and youth care workers), and service areas (ie, ambulatory and intensive services).

#### KMS Implementation

Testers were onboarded by watching a 15-minute introduction video and attending a 30-minute web-based session demonstrating KMS functionalities. The introduction video provided the rationale for KMS, which included sharing examples intended to illustrate the value proposition of the tool as a “one stop shop” for finding information. The development of the KMS was also described, including the role of stakeholders. After the demonstration, the testers participated in a hands-on practice exercise by completing a scavenger hunt. Testers were incentivized to return their scavenger hunt by being entered into a draw to win 1 of 3 coffee cards worth CAD $10 (US $7.50). Testers were provided with access to the KMS over a period of 10 weeks and were instructed to use the system as required to complete their daily work (eg, use the system to access documentation forms and to find information on community resources for their clients). Project leads were available for individual consultation or coaching during the pilot evaluation. Reminders regarding the use of KMS were provided at biweekly intervals, and tip sheets on various KMS functionalities were shared via email.

#### Data Collection and Analysis

##### System Use Analytics

System use analytics were tracked throughout the pilot and reported for weeks 2 to 10 (week 1 was excluded from analyses as testers were still being onboarded). Analytics reported include the total number of content views per week across all testers, total days active across all testers, percentage of active testers per week (ie, the proportion of testers who viewed or downloaded, at least once, a piece of content in the week or number of testers who accessed the KMS after training), and most viewed pieces of content.

##### Survey

The testers completed surveys before being introduced to the KMS (baseline) and at the end of the 10-week pilot-test (end of pilot).

The baseline and end-of-pilot survey asked testers to rate their current level of satisfaction by accessing six different types of content: (1) content used in clinical sessions; (2) content used for clinical learning; (3) information on MHA-specific education and training; (4) MHA program and operations; (5) information on community resources, events, and programs; and (6) MHA documents that were currently organized on an “MHA standard work dashboard.” The standard work dashboard is an internally developed information management tool that is available at the baseline to organize access via hyperlinks to a specific type of content to provide easy access and support continuous improvement efforts (standard work and fillable forms). Satisfaction with accessing each type of content was rated on a 5-point Likert type scale with anchors of “very dissatisfied,” “dissatisfied,” “neutral,” “satisfied,” and “very satisfied.”

The end-of-pilot survey included additional items asking testers to rate KMS functions, including overall ease of use, relevance or quality of content, ability to share content, discover new content, ease of downloading content, and save time finding information. Ratings were provided on a 5-point Likert scale with anchors of “poor,” “fair,” “good,” “very good,” and “excellent.” The testers also indicated whether they would recommend the KMS to a colleague using the following options: “definitely,” “probably,” “not sure,” “probably not,” and “definitely not.”

Survey responses were reported as frequencies and percentages. For satisfaction with accessing content, the frequency and percentage of respondents who indicated that they were either satisfied or very satisfied were reported at baseline and after the pilot. Notably, because 2 types of content were relevant only for testers who were clinical staff (content used in clinical sessions and content used for clinical learning), testers who were administrative staff were excluded from reporting on these items. For ratings of KMS functionalities, the frequency and percentage of respondents who indicated “very good” or “excellent” were reported. Frequencies and percentages of respondents providing all responses were reported for the item on recommending KMS to a colleague.

##### Focus Groups

At the completion of the pilot study, testers were invited to participate in 1 of 3 focus groups. Focus groups were conducted by a member of the evaluation team (DJE) with support from a research assistant (OR) and lasted approximately 45 minutes. The questions covered four broad areas: (1) testers’ experiences with the KMS, (2) their use of the KMS, (3) feedback on training, and (4) advice for implementation. See Multimedia Appendix 1 for the detailed questions. All focus groups were transcribed and analyzed using thematic analysis, as outlined by Braun and Clarke [13]. Analyses were completed by an initial reviewer (DJE) who reviewed the transcripts to create initial high-level themes with a summary and exemplar quotes. The second reviewer (JC) further refined and categorized the themes. The themes were reviewed and validated by the KMS evaluation and steering committees.

## Results

### Recruitment

Of the 33 individuals who expressed interest in becoming a tester, 30 testers ultimately completed the training. The testers included 22 interprofessional care providers (including 5 social workers, 3 youth or transition care workers, 7 registered nurses, 4 psychologists, 1 psychiatrist, 1 recreation therapist, and 1 occupational therapist) and 8 administrative staff (including 1 group coordinator, 2 booking and registration clerks, and 5 administrative assistants) and represented a range of service areas (ambulatory, inpatient, and day treatment).

### System Use Analytics

**Figure 1:**
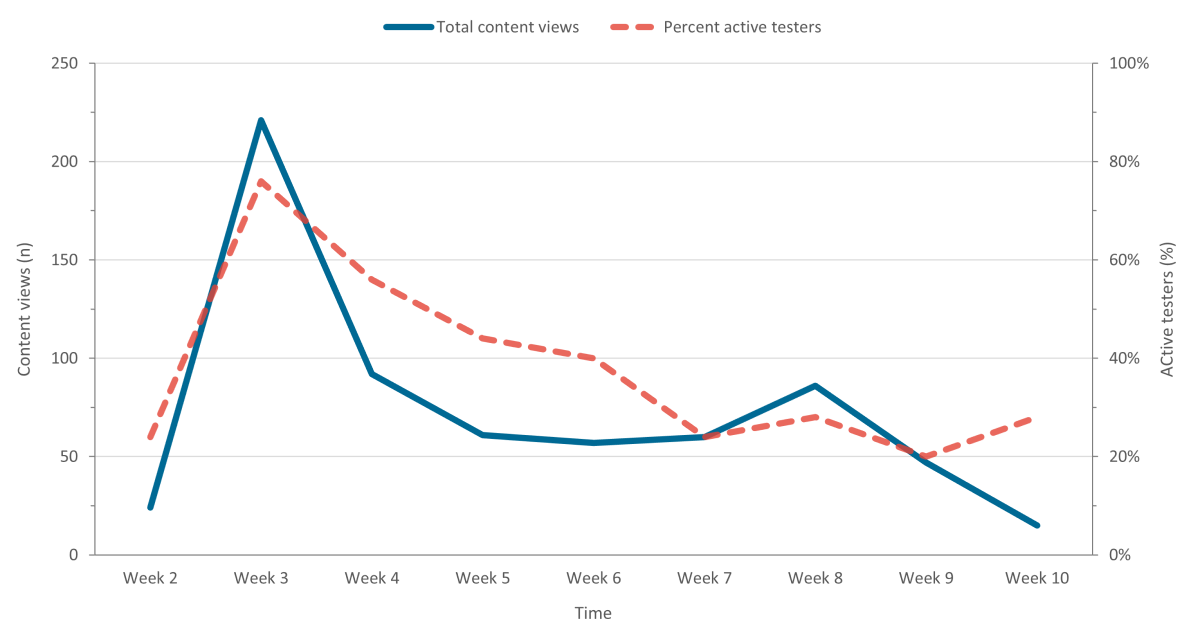
Weekly content views and percent active testers on the knowledge management system platform over the test period.

Content views and the percentage of active testers over the course of the pilot study are shown in Figure 1. Of the 30 testers who were trained, 25 (83%) accessed the KMS at least once after their initial training and scavenger hunt. A median of 7 testers were active each week, and these testers were active for a median of 4 days over the course of the pilot (range 1-17 days) study. Activity was the highest in the third week (221 content views across 19 testers) and decreased across the pilot study. A median of 21 pieces of content were viewed by each user during the pilot (range 1-150) study, with clinical staff generally viewing more content (median 24) than administrative staff (median 3). The 10 most viewed pieces of content were fillable forms used for clinical documentation and content related to the KMS (ie, tips for KMS testers and scavenger hunt).

### Survey

In total, 27 of the 30 trained testers (21 clinical care providers and 6 administrative staff) returned baseline surveys, and 26 of the 30 trained testers (22 clinical care providers and 4 administrative staff) returned the end-of-pilot surveys.

**Figure 2:**
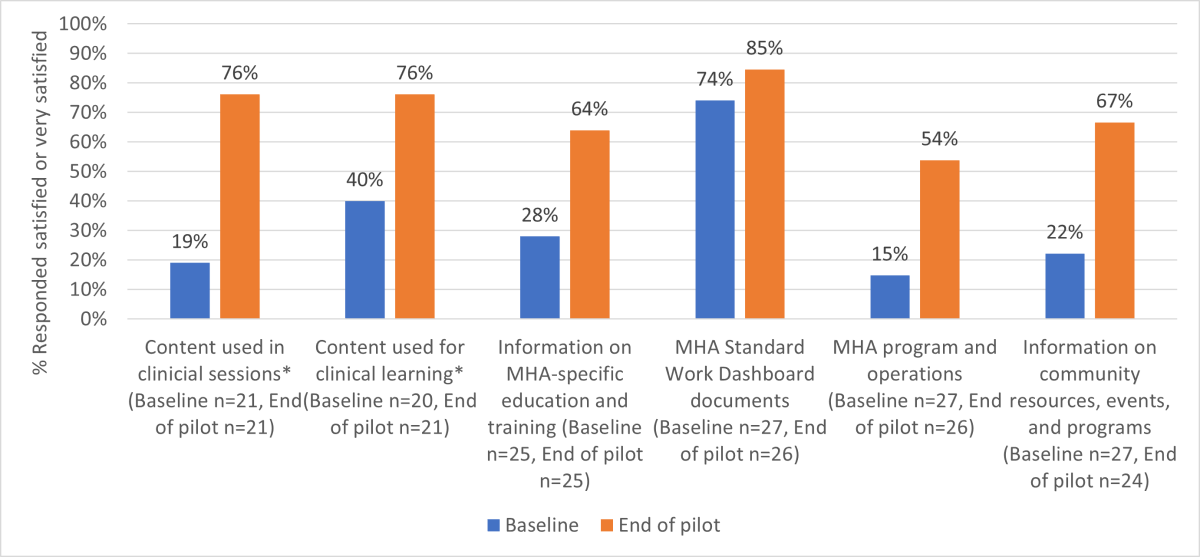
Ratings of satisfaction with accessing types of content at baseline and end of pilot (*includes only clinical staff participants). MHA: mental health and addictions.

Figure 2 displays the percentage of respondents who were “satisfied” or “very satisfied” with accessing the types of content at baseline and end of pilot. Overall, there were increases in satisfaction with the KMS content over previous methods across all areas surveyed, with the largest increase in satisfaction occurring for content used in clinical sessions (57% increase) and the lowest in the MHA standard work and dashboard documents (11%), which had a high level of satisfaction at baseline.

In terms of ratings of KMS functions, 82% (18/22 respondents) rated quality of content and ease of use as very good or excellent. Ease of downloading content was rated by 79% (15/19 respondents) as very good or excellent. Ability to share content and discovering new content was rated by 78% (14/18 and 17/22 respondents, respectively) as very good or excellent. Relevant Content was rated by 75% (15/20 respondents) rated relevant content as very good or excellent. Saving Time Finding Information was rated by 67% (14 of 21 respondents) as very good or excellent.

In total, 79% (19/24 respondents) of testers indicated that they would “definitely recommend,” 13% (3/24 respondents) of testers indicated they would “probably recommend,” and 2 testers indicated they were “not sure” if they would recommend the KMS to a colleague. No testers indicated that they would “probably not” or “definitely not” recommend the KMS.

### Focus Groups

A total of 17 testers participated in the 3 end-of-pilot focus groups. The themes are described in subsequent sections.

#### Theme 1: The KMS Helps Solve a Problem for Staff

Testers participating in the focus groups noted that KMS was a solution to the problems they currently experience in finding and storing information. The ability to have a central place to store information, particularly content related to community resources and events, was noted as a key benefit of the KMS. Before the KMS, this type of information was generally sent as email notifications, and having a central location where testers knew this information would be stored could result in “guilt free [email] deleting.” The benefit of a KMS over current storage solutions was also commonly referenced as “It’s better than the [shared] drive.” Finally, focus group testers commented on the benefits of reducing cognitive load. Knowing that all information was on the KMS freed up cognitive space that used to be spent recalling where a piece of information was received from and where it had been stored (eg, in their email, shared drive, or filing cabinet). One tester shared a selling feature of the KMS platform as “...all in one, rather than going to all the drives.”

#### Theme 2: Functionality of the KMS Is Important

The focus group testers indicated an overall positive experience with the KMS. The intuitive ease of use of the platform itself was noted as a key feature that contributed to positive experiences. For example, testers commented on the utility of the search feature, their appreciation for being able to see what others are using, and the benefit of the system generated recommended related content, such as “I like the Netflix feature, if you looked at this content, then you might be interested in this content shows up.” In addition, testers appreciated the ability to share content with their colleagues, “there’s peace of mind that the information is there...nice to be able to share with colleagues.” However, one of the features of the platform created a minor challenge for users. The KMS allows users to filter content, but these filters must be cleared when performing a new search. Focus group testers found that they often forgot to clear filters, resulting in them not finding content when it was available on the KMS.

#### Theme 3: Quality Content Matters

The focus group testers identified the content as the primary driver of using the KMS. The variety of the available content was appreciated, with focus group testers noting that they accessed clinical documentation, handouts, worksheets, and videos. They described having valid, reliable, curated, and current content as a key driver of their KMS use. One tester stated, “I don’t need to read the whole document before I give it to my clients, I can trust the information,” and another shared, “...KMS is far more geared to what I am looking for, it is accredited and I trust what I pluck off there I can just use.” Having robust and relevant content was highlighted as being particularly important for KMS use. For example, the absence of medication information sheets was a significant gap for psychiatry and administrative staff, who noted that most of the content did not apply to their roles. When focus group testers could not find the relevant content, they would revert to the past practices.

#### Theme 4: Training Was Helpful and Could Be Improved

Testers in the focus groups found the training format helpful, particularly the use of incentives (eg, gift card draw) to complete the scavenger hunt to encourage engagement with the platform for the initial introduction. Testers in the focus groups also commented on the importance of having an identified person (ie, the project management lead) to reach out to with questions and that receiving general tip sheets was beneficial. That said, testers in the focus groups recommended a more distributed practice over the course of the pilot; they commented that having too much upfront meant they lost some of the key training points as they were not yet actively using the platform, with 2 testers noting, “I don’t remember anything from the training,” and another noting, “I needed to play around with it,” rather than spending time in a training session.

#### Theme 5: Make Accessing the KMS Easy and Barrier Free

Focus group testers noted that getting to the log-in page and the log-in process as 2 access barriers. For example, there were multiple clicks required to get to the log-in page; this was addressed by sending out a desktop icon to permit ease of navigation to the KMS. Although log-in is only required once every 7 days, for those new testers or those who used the KMS infrequently, the need to log-in for each use was a barrier. For example, 1 tester stated, “It’s just easier to go to where I know I have the resource than to click and log-in to the KMS and then search from something I already have bookmarked [in my Internet browser].” Consistent with the observation that accessing information from current (ie, non-KMS) sources was a habit, testers recommended that access to content through other routes (eg, shared drive folders) should be removed.

## Discussion

### Principal Findings

This paper describes a quality-improvement project to develop a KMS and pilot-test its implementation in 1 MHA program. A commercially available electronic knowledge management platform was procured. Stakeholder engagement was used to identify the content and draft the initial information architecture and content management processes. KMS use and satisfaction were assessed during the pilot evaluation. KMS use was the highest in the third week and decreased across the pilot evaluation. Approximately one-third of the testers were active each week. Despite decreases in use over the course of the pilot study, the results indicated that testers found the KMS to be a useful tool. The ratings of satisfaction with accessing the content increased from the beginning to the end of the pilot. Approximately three-quarters of the testers were satisfied with the various functions of the KMS, and most testers would definitely or probably recommend the KMS to a colleague. The results of qualitative focus groups indicated that testers found that the KMS solved a problem for staff, that the functionality and quality of content in the KMS are important, and that training and access to KMS could be improved.

Our KMS development and implementation process was designed to address several key success factors identified in knowledge management literature [14-17]. The ease of use and quality of technology have consistently been identified as facilitators of the uptake of information technology solutions [14,17]. In our case, procurement of a commercially available platform resulted in a highly usable solution that allowed us to capitalize on advanced features, such as predictive searching and suggested related content. Leadership support and dedicated resources have also been identified as key to the success of knowledge management initiatives [18,19]. In our work, we addressed this contributor by tasking project and clinical leads with overseeing the project, working with the direct support of an executive level steering committee, and providing dedicated time for testers to engage in training and practice. In addition to instructions on how to use the system, our training materials explicitly outlined the value proposition (ie, a “one stop shop”) and stakeholder engagement in KMS development. This communication was intended to increase motivation, as beliefs about benefits and motivation have also been highlighted as contributors to knowledge management and technology adoption [10,18,20]. Finally, coproducing knowledge management strategies and procedures have also been recommended in the knowledge management literature, and we addressed this by engaging stakeholders throughout the project [18].

The results of our pilot evaluation supported the assertion that there is a need for knowledge management in MHA services. At baseline, with the exception of content that was already curated on an internal dashboard, satisfaction with accessing the content was low. Indeed, only about a quarter of testers were satisfied with their current methods of accessing information. Our organization is not unique in this regard, with knowledge management being highlighted as a key challenge in health, mental health, and social services agencies [20,21]. For example, the results of a qualitative study with health care leaders in Ontario, Canada, suggested that despite extensive knowledge needs, leaders universally agreed that there was a paucity of systematic knowledge management and that information tools were not available or did not meet their needs [9].

Despite initial challenges in accessing content, our results provide evidence that a KMS is a promising solution to improve knowledge management in MHA. Satisfaction with the ability to access content improved from the beginning to the end of the pilot, and testers rated the functionalities of the KMS highly. These findings were similar to those of a pilot evaluation of an electronic KMS (WAX Active Library) in general medical practice [22]. In their study of 19 general practice physicians, O’Brian and Cambouropoulos [22] found that 90% of users rated KMS easy to use, and all users found the system easy to navigate. All the physicians indicated that they wanted to continue using the system after the study. In another study, Meenan et al [23] implemented a Wiki format as a KMS in a radiology information technology department. The results indicate that users engaged in the Wiki format, spending an average of 5 hours per week using Wiki to address these issues.

Although the ratings of satisfaction were high, our results suggest that ongoing KMS use was a challenge, with only a small proportion of testers active per week. This is not surprising, given that ingrained patterns of behavior are difficult to change, and motivation to adopt KMSs has been identified as a key challenge in the literature [24]. Indeed, other authors [25,26] have reported on challenges with sustaining knowledge management efforts. Our findings are discrepant with those of O’Brian and Cambouropoulos [22] and Meenan et al [23], which indicated that KMS use was sustained over the test period or even increased. Although we attempted to address the barriers and facilitators in our implementation plan, this evidence suggests that further work is required. Future research and implementation efforts should more thoroughly examine the predictors of KMS use [11,14,16,27]. For example, KMS content quality has been found to predict user satisfaction and perceived usefulness [19]. The results of our qualitative focus groups suggested that there were content gaps in our pilot version, particularly with regard to administrative content, and this may have affected KMS use.

Qualitative feedback highlighted important considerations for the continuous improvement of the KMS design and implementation. As described earlier, the focus group findings indicated that there was missing content related to administrative staff and the need for psychiatrists. Since this project, we have iteratively added content, including medication handouts commonly used by psychiatrists, and identified additional operational content needs such as contact lists, links to organization-wide information (eg, finance forms), and letter or email templates. The qualitative results also highlight potential improvements in training and implementation planning. There was feedback related to increasing opportunities for distributed practice, ensuing a dedicated support person was identified, and addressing access issues (eg, integrating with institutional log-in, removing content access through existing mechanisms). These recommendations were also actioned because this project included extending practice opportunities and introducing a dedicated role of the “KMS Champion” within teams. To facilitate access, the log-in to the KMS platform was integrated with institutional credentials, and a desktop icon linking to the platform was also pushed to all computers.

This study has some limitations. Given that the study was conducted primarily as a quality-improvement project, our approach was pragmatic, leading to limitations. For example, the limited sample size and missing data limited our ability to statistically test changes over time or the effects of user type or service area. Furthermore, given that this was not an experimental study, we cannot conclude that changes from before to after the pilot study were a result of the KMS. Although we purposefully sampled to include a range of roles and professions that participate as testers, we do not have any additional information on their demographic characteristics or their level of comfort with technology. Thus, it is difficult to comment on the degree to which they were representative of the full population of staff in the MHA program. This project also did not comprehensively hypothesize or collect data on the predictors of KMS use or satisfaction. There are current theoretical models and empirical studies on predictors of knowledge management success [14-17,27-29] and uptake of technology [11,30,31]. Future research could more thoroughly apply this work to identify evidence-based predictors of KMS uptake, so that they can be targeted in future interventions. Furthermore, this project could have made a greater contribution to the literature by testing a theory of change or being tied explicitly to implementation science methods [32]. Finally, although we use the term knowledge management throughout this paper, we recognize that, to date, our project is more consistent with information management, as we have included only explicit knowledge captured through various types of documentation.

### Conclusions

Providing high-quality, efficient, and timely MHA care requires ready access to a wide range of knowledge, tools, and resources. Knowledge management is an important need in mental health, and addictions and technology-enabled KMSs hold promise for addressing this need.

This paper reports on a quality-improvement project to develop a KMS and pilot-test its implementation in 1 MHA program. The KMS used stakeholder engagement to design its architecture and supporting processes. The results of a pilot evaluation indicated that users were satisfied with the KMS functionalities, and most testers would recommend the KMS to a colleague. That said, KMS use decreased over the course of the pilot, and the results of the focus groups recommended improvements to KMS content and implementation processes. To our knowledge, this is one of the first studies to report the development and implementation of a KMS in 1 MHA program. By reporting on our KMS development and collecting feedback on the implementation process, this project can provide guidance to other organizations conducting similar work. Future research could more systematically examine the predictors of KMS use, test the efficacy of additional implementation strategies, and eventually examine whether implementing a KMS improves the health system (eg, capacity and efficiency) and patient outcomes.

## Appendix

Multimedia Appendix 1 Focus group question guide.

## Abbreviations

KMS: knowledge management system
MHA: mental health and addictions

## Data Availability

The data sets generated and analyzed during this study are available from the corresponding author upon reasonable request.
