# Supplementary material for: Implementation of a Knowledge Management System in Mental Health and Addictions: Mixed Methods Case Study
Source: JMIR Form Res. 2023 Feb 6;7:e39334. doi: 10.2196/39334 (PMC9941906; doi:10.2196/39334)
Supplement: Multimedia Appendix 1 [file formative_v7i1e39334_app1.docx]

Multimedia Appendix: Focus Group Question Guide

1. Experience with KMS

- Can you tell us about your experience with the KMS?
- What difference did the KMS make in your day-to-day work?
- What do you like most about the KMS?
- What would you most like to change about or add to the KMS?

1. Use of the KMS

- What types of information did you come to the KMS for?
- What wasn’t there that be helpful to have there?
- How often should people be coming to the KMS?

1. Feedback on the training

- What are some of the key points you remember from or you returned to from the training?
- What was good about the training?
- What could be improved?
- Sharing tips or tricks; how much time would be needed to be able to use the KMS?
- What supports were helpful?

1. Advice for implementation

- What would be important for us to be doing, considering, thinking about when planning for roll out across the program?
- What would improve adoption/use?
- What is the role of leadership?
